# A Study on Perception and Exposure to Occupational Risks at Public School Food Services in Bahia, Brazil

**DOI:** 10.3389/fpubh.2022.891591

**Published:** 2022-06-09

**Authors:** Jeane dos Santos Ferreira, Maria da Purificação Nazaré Araújo, Raquel Braz Assunção Botelho, Renata Puppin Zandonadi, Eduardo Yoshio Nakano, António Raposo, Heesup Han, Marcelo Arraño Muñoz, Antonio Ariza-Montes, Rita de Cássia Coelho de Almeida Akutsu

**Affiliations:** ^1^School of Nutrition, Federal University of Bahia, Salvador, Brazil; ^2^Department of Nutrition, Faculty of Health Sciences, University of Brasília, Brasília, Brazil; ^3^Department of Statistics, University of Brasília, Brasília, Brazil; ^4^CBIOS (Research Center for Biosciences and Health Technologies), Universidade Lusófona de Humanidades e Tecnologias, Lisboa, Portugal; ^5^College of Hospitality and Tourism Management, Sejong University, Seoul, South Korea; ^6^Facultad de Ciencias Sociales, Universidad Autónoma de Chile, Santiago, Chile; ^7^Social Matters Research Group, Universidad Loyola Andalucía, Córdoba, Spain

**Keywords:** low-income, food handlers, occupational risks, school food services, Brazil

## Abstract

Food service work is hazardous due to the intense rhythm of food production, and the working conditions can cause discomfort, fatigue, and occupational accidents and illnesses. For the perception of exposure to occupational hazards, workers must participate in continuing education programs. This study aimed to verify the perception and exposure to occupational risks at school food services (SFS) in Bahia, Brazil. This cross-sectional study was conducted in SFS from public schools in Bahia/Brazil. Researchers identified sociodemographic variables, occupational characteristics, and the Perception of Exposure to Occupational Risks by SFS food handlers. Also, anthropometric assessment (weight, height, and waist circumference), the presence of comorbidities, and the identification of exposure to occupational risks and measures of environmental comfort were evaluated. Most workers were female (98.6%; *n* = 140), mean age of 46.85 y/o, working as SFS food handlers between 1 and 5 years (50.7%; *n* = 72) but with no training on occupational risks (52.8%; *n* = 75). This lack of training is not associated with demographic or other occupational variables. The majority of the food handlers present a fair or good perception of exposure to occupational risk. These food handlers are also mostly overweight, and higher BMI was associated with hypertension and edema. The SFS were classified as of high occupational risk (mean of 31.24% of adequacy) environments. Ergonomic Risks had the lowest percentage of adequacy (7.69%, very high risk) regarding occupational risks, followed by chemical risks (31.5% of adequacy, high risk), accident risk (32.19%, high risk), and physical risk (36.89%, high risk). The excess of activities associated with precarious physical structure, insufficient number of equipment and utensils (in inadequate conservation) favors the exposure to occupational risk in SFS.

## Introduction

Occupational hygiene is the science that studies workers' exposure to occupational risks and suggests control measures to prevent health problems in work activities (1). Occupational risks refer to the likelihood that an injury or illness occurs due to exposure to workplace hazards. Such risks are classified as physical, chemical, biological, ergonomic, and mechanical, according to their cause. They can occur in different work environments (industries, constructions, hospitals, and food services). Among them, food services have been studied since they are an important economic segment that plays an essential role in the individuals' health and well-being through the quality of the produced food, influencing aspects of public health, society, and the environment (2).

Food service work is hazardous due to the intense rhythm of food production and high environmental temperature, noise, and humidity; activities with risk of injury; pressure for delivery of meals on time; insufficient number of equipment, and inadequate physical structures (3). These working conditions can cause discomfort, fatigue, and even occupational accidents and illnesses (4). Food services have accident and illness rates like industries commonly recognized as hazardous workplaces (5). In Brazil, educational food services are important for the employment of food handlers since adequate food is considered a right for students enrolled in public schools through the National School Feeding Program (PNAE) (6). It is essential to highlight that, unlike in other countries, school meals in Brazil are the right of every student enrolled in basic education. PNAE is considered one of the world's most significant and long-lasting government programs in school meals (7) offering meals at public schools to low-income students (8–10). According to the number of students enrolled in each school system, the Brazilian Federal Government transfers supplementary financial amounts to states, municipalities, and federal schools in 10 monthly installments (from February to November) to cover 200 school days. Currently, the amount transferred by the Union to states or municipalities per school day for each student is defined according to the stage and type of education (daycare centers: R$ 1.07; preschool: R$ 0.53; indigenous and quilombola community: R$0.64; elementary and secondary education: R$0.36; full-time education: R$1.07) (11). However, this federal government's investment is meager (12), and the funds are just enough to purchase food staples. Thus, municipalities and states are responsible for school kitchen infrastructure, which is not always a priority for them. Therefore, occupational risks are also found in School Food Services (SFS), and recognizing them, allows the adoption of preventive/corrective measures to minimize/eliminate them in the work environment (13). In PNAE, the nutritionist is responsible for planning, coordinating, directing, supervising, and evaluating all food and nutrition actions within the scope of school meals (11), being responsible for food handlers.

Navarro and Cardoso (14) propose the concept of risk perception as a phenomenon processed with an awareness of self-perception, integrated into a specific collective context that encompasses a behavioral perspective, also associated with personal factors related to the capacity of cognitive conception, to affective and biological aspects and as possibilities of interaction with the external environment. According to social psychology, perception captures external stimuli by the senses and the organization and interpretation of these experiences by cognition. Thus, occupational risks must be identified by social networks through risk perception. For the perception of exposure to occupational hazards, workers must participate in continuing education programs to identify factors related to exposure and use control measures to minimize and/or eliminate occupational hazards (1). In this sense, this study aimed to verify the perception and exposure to occupational risks at SFS in Bahia, Brazil.

## Materials and Methods

### Study Design

This study is part of a broader research project entitled “Occupational Risks in School Food Services,” approved by the Research Ethics Committee of the School of Nutrition of the Federal University of Bahia (protocol n° 2,121,882).

A cross-sectional, exploratory study was conducted in SFS from Salvador/Bahia/Brazil public schools. Salvador/Bahia, is considered the center of Afro-Brazilian culture, with most of the population (about 80%) black or brown (15). It was the first Brazilian capital and is one of the largest cities in the country, with a population of around 2.9 million inhabitants (16). It has great social inequality and suffers from unemployment, violence, poor health, and uncontrolled growth. In the 1950s and 1970s, the municipality experienced intense growth, with the massive occupation of peripheral areas and the peninsula's center by the socially and economically vulnerable and black population (15). Current patterns of space appropriation and racial and social segregation in the municipalities of Salvador were defined and consolidated from 1960 onwards and, consequently, social vulnerability.

Data collection occurred from August 2018 to August 2019. It included: (1) identification of exposure to occupational risks and measures of environmental comfort: humidity, temperature, noise, and luminescence in SFS (17); (2) identification of sociodemographic, occupational characteristics, and the Perception of Exposure to Occupational Risks by SFS food handlers (17) (3) anthropometric assessment and presence of comorbidities in food handlers (17).

### School Food Services Sampling

At the beginning of the project, 434 public municipal schools were functioning and could participate in the study. We used a sample calculation with a confidence level of 95% and an error of 5%, which resulted in a sample of 159 schools (35.6% of the total), randomly selected by stratified probability sampling based on the number of existing schools using Excel® 2005 program for Windows 10 version. All schools were included in the random draw (each school received a number before the draw). If the school's principal did not agree to participate in the study, the immediately following school was invited to participate. Only three principals did not agree to participate, and all the subsequent schools' principals agreed to participate in the study. The municipal schools included in the sample were not identified for ethical reasons.

### Exposure to Occupational Hazards and Environmental Comfort Measures (SFS Humidity, Temperature, Noise, and Luminescence)

To evaluate the exposure to occupational hazards and environmental comfort measures (SFS humidity, temperature, noise, and luminescence), an instrument previously validated by Ferreira et al. (17) was used. The instrument consisted of 97 items distributed in five Thematic Blocks (TB) (17): (i) Physical risks, with 28 items. (ii) Chemical risks (12 items); (iii) Biological risks (13 items); (iv) Ergonomic risks (14 items); and (v) Mechanical (accident) risks (30 items) (17).

The forms were filled out by the researchers using a three-point scale: Yes (1 point) for the item conforming to the Brazilian legislation; No (0 points) for the item that does not attend to Brazilian legislation and is Not applicable, when the item did not apply to SFS (18). The adequacy of exposure to occupational risks was evaluated through the applicable notes of TB obtained through Equation 1:


(1) $$\begin{array}{l} \;Exposure\;to\;occupational\;risk=\\ \frac{number\;of\;''yes^{\prime \prime }-''not\;applicable^{\prime \prime }\;items}{Total\;of\;item\;from\;thematic\;block\;}x\;100 \end{array}$$

The exposure to occupational risks in the SFS classification was based on the sum of the applicable notes of the TB. The result was presented as a percentage of compliance. For the final classification of each SFS, Equation 2 was used:


(2) $$\begin{array}{l} \;Classification\;of\;exposure\;to\;occupational\;risks\;=\\ \frac{\left(\sum Number\;of\;''yes^{\prime \prime }-\;\sum ''not\;applicable^{\prime \prime }\;items\right)}{Total\;of\;instruments'items\;}x\;100 \end{array}$$

The research classified the exposure to occupational risks as Very High Occupational Risk Situation (0 to 25%); High Occupational Risk situation (26 to 50%); Regular Occupational Risk (51 to 75%); Low Occupational Risk (76 to 90%) and Very Low Occupational Risk (91 to 100%) (19, 20).

### Identification of Sociodemographic and Occupational Characteristics and Perception of Food Handlers Perception of Exposure to Occupational Risk

The sample size calculation considered the number of food handlers (*n* = 318) working in the 159 randomly selected schools to participate in the study. A minimum sample of 139 food handlers was calculated using a confidence interval of 95% and a sampling error of 5%. Food handlers were randomly selected within geographic areas from Bahia/Brazil. There was no refusal from randomly selected food handlers, and three of the unselected food handlers voluntarily requested inclusion in the study. Thus, 142 food handlers participated in this phase.

The identification of sociodemographic and occupational characteristics and the Perception of Exposure to Occupational Risk by food handlers occurred through a semi-structured questionnaire previously validated (17). The questionnaire consisted of 32 items, distributed in thematic blocks: (1) socioeconomic characteristics (17); (2) information about the work routine (17); (3) Perception of Occupational Risks, with items that integrate the guidelines of the Regulatory Standards of the Brazilian Ministry of Labor: NR 6, NR 9, NR 17, and NR 24 (21–24).

The items on the perception of exposure to occupational risk form were evaluated using a five-point Likert scale, ranging from: (1) I totally disagree, (2) I partially disagree; (3) I neither agree nor disagree; (4) I partially agree; (5) I totally agree. Thus, the handlers' perception was classified as: Very Low Perception (0 to 1.5); Low Perception (1.51 to 2.5); Regular Perception (2.51 to 3.5), High Perception (3.51 to 4.5), and Very High Perception (4.51 to 5) (17).

### Anthropometric Assessment and Presence of Comorbidities in Food Handlers

A form was used for the anthropometric assessment and comorbidities in food handlers. The data collected were: sex, age, weight, height, waist circumference, presence or absence of comorbidities, such as arterial hypertension, diabetes mellitus, dyslipidemia, hyperthyroidism or hypothyroidism, mycoses, dermatitis, edema, headache, and musculoskeletal problems. The researchers verified the presence of edema, weight, height, and waist circumference (WC), and the handlers provided the other information.

A digital scale (G-TECH®) was used to check body weight with a maximum capacity of 180 kg and a precision of 100 grams. The handlers were weighed barefoot, wearing light clothes, and instructed to remove heavy objects, such as keys, belts, glasses, cell phones, and any other materials in their pockets or items that could interfere with their body weight (25). Pregnant women, if randomly selected, would be excluded from the sample due to changes in anthropometric measurements during pregnancy. However, none of the randomly selected individuals was pregnant.

Height was verified using a vertical stadiometer (Compacto E210-Wiso^®^) ranging from 0 to 180 cm, precision of 1 mm, fixed on a flat surface, keeping the red line at landmark 0. The handler was positioned standing, leaning against the wall (25). The head was positioned in the Frankfurt plane (the inferior margin of the orbital opening and the superior margin of the external auditory meatus were in the same horizontal line). Weight and height measurements were used to calculate the body mass index (BMI) according to the equation: BMI = weight/height^2^. The nutritional status of food handlers was classified according to the World Health Organization (WHO) recommendation for adults and the elderly: (1) Low weight (BMI <18.5 kg/m^2^); eutrophy (BMI ≥ 18.5 to 24.99 kg/m^2^); overweight (BMI ≥ 25.0 to 29.99 kg/m^2^) and Obesity (BMI ≥ 30 kg/m^2^) (26).

An inextensible and inelastic measuring tape was used to measure the waist circumference, with a scale of 0.5 cm, placed at the midpoint between the last rib and the iliac crest in a horizontal plane (25). Waist circumference classification (WC) corresponded to the following values: level 1, (WC) between 80.0 and 87.9 cm for females and between 94.0 and 101.9 cm for males; level 2, (CC) greater than 88.0 cm and 102.0 cm for females and males, respectively. Given the above, descriptive statistics were differentiated between the two groups, data from male handlers was excluded for the analysis of the relationship between WC and the presence of comorbidities.

### Data Processing and Analysis

The data were entered into a specific database created in the Statistical Package for Science Program-SPSS^®^, version 26.0, in which the analyzes were also processed. After creating the data entry form, a check was carried out through frequency distribution analysis, comparing the values of each variable in the SPSS^®^ database with those possible to occur, avoiding typing errors. To analyze the relationship between BMI and WC with other variables, male handlers were excluded due to the size of this sample (two individuals). For the characterization of the sample, the following were used: frequency, mean, percentage, and standard deviation. Means differences were verified by One-way analysis of variance (ANOVA) followed by Tukey's *post hoc* test and unpaired Student *t*-test. For analysis of proportions, Pearson's chi-square test was used. All tests were performed considering the bicaudal hypothesis and a 5% significance level.

## Results

### Identification of Exposure to Occupational Risk and Measures of Environmental Comfort: Humidity, Temperature, Noise, and Luminescence

Table 1 presents the Thematic Blocks, percentage, and classification of Exposure to Occupational Risk in SFS. The TB that assesses exposure to ergonomic risks was classified as Very High Occupational Risk (0 to 25% adequacy), and the other TB as High Occupational Risk (26 to 50% adequacy). The overall classification of the SFS corresponded to High Occupational Risk (26 to 50% adequacy). In TB 1 (Physical Risks), the assessment of temperature, ambient humidity, and noise occurred in the physical or technical areas of the SFS (**Table 6**). The factors that contributed to the classification were: temperature >26.7°C in the pre-preparation/cooking areas. It is noteworthy that in most SFS (>90%), there was no separation between the pre-preparation/cooking areas. Other factors related to workers' exposure to occupational hazards were inappropriate location and configuration (86.2%; *n* = 137). Such inadequacy compromises thermal comfort and excessive steps to perform handlers' tasks (48.4%; *n* = 77).

**Table 1:** Thematic Blocks, percentage and classification of Exposure to Occupational Risk in school food services.

**Thematic Blocks**	**% of conformity**	**Classification of exposure to occupational risk**
Physical risks	36.89	High
Chemical risks	31.50	High
Biological risks	34.93	High
Ergonomic risks	7.96	Very high
Accident risks	32.19	High
Mean	31.24	High

In TB 2 (Chemical Risks) non-conformities were identified: non-use of PPE, such as polyvinyl gloves in 87.4% (*n* = 139) and polycarbonate glasses in 99.4% (*n* = 158) for dilution/use of chemical products recommended for cleaning the areas. Inadequate storage of hygiene products, in areas intended for food manipulation, occurred in 39.6% (*n* = 63) of SFS. TB 3 (Biological Hazards) were identified: the storage of vegetables and fruits not sanitized in the pre-preparation/food preparation area in 61.6% (*n* = 98); presence of vectors and urban pests in 89.3% (*n* = 142); trash bins without pedal activation in 62.3% (*n* = 99); non-adequate hand hygiene of food handlers in the pre-preparation, preparation, and distribution of food in 74.8% (*n* = 119); 98.7% (*n* = 157) did not use polyvinyl gloves for cleaning vegetables and fruits and for disposing of food in diners' utensils.

TB 4 (Ergonomic Risks) had the lowest percentage of adequacy. Among the factors that contributed to the low adequacy were: lack of labor gymnastics (100%; *n* = 159); absence of a complete uniform (74.2%; *n* = 118) for the thermal comfort of the handlers; non-use of lumbar protection vests (99.4%; *n* = 158) during weight-bearing; absence of semi-sitting benches and support for footrest (93.1%; *n* = 148) and absence of transport cars (96.9%; *n* = 154). In TB 5 (Accident Risks), it was identified that the equipment in 65.4% (*n* = 104) and the presence of "support columns, in the center of the areas, in 34% (*n* = 54), reduced the space for the circulation of people. Also, the absence of extinguishers or extinguishers with an expiration date in 81.8% (*n* = 130) and 100% of the SFS for class K extinguishers, intended to fight the flames of food preparation that use flammable oils and fats.

Table 2 presents the mean and standard deviation of environmental comfort measures in the SFS areas. The averages of humidity and noise are according to the Brazilian Ministry of Labor (27).

**Table 2:** Mean and standard deviation of environmental comfort measures in the areas of the SFS (Bahia/Brazil).

**Areas**	**Environmental comfort measures**	**Mean**	**SD**
Storage area	Temperature (°C)	25.29	1.49
	Humidity (%)	67.1	7.54
Pre-preparation area	Temperature (°C)	26.28	1.51
	Humidity (%)	68.39	1.22
	Noise (dB)	70.33	6.04
Cooking area	Temperature (°C)	27.21	2.36
	Humidity (%)	62.09	9.5
	Noise (dB)	71.30	6.79
Distribution area	Temperature (°C)	25.23	1.61
	Humidity (%)	65.70	8.52
	Noise (dB)	79.79	7.91
Utensils' cleaning area	Temperature (°C)	25.64	1.55
	Humidity (%)	69.01	8.04
	Noise (dB)	70.66	6.39
**Total**	Temperature (°C)	25.93	1.16
	Humidity (%)	67.55	2.53
	Noise (dB)	73.03	4.53

### Identification of Sociodemographic and Work Characteristics and Perception of Exposure to Occupational Risks by Food Handlers at SFS

Table 3 presents the sociodemographic and work characteristics of the SFS food handlers who participated in the study. Most were female (98.6%; *n* = 140), the mean age was 46.85 y/o, working as SFS food handlers between 1 and 5 years (50.7%; *n* = 72) but with no training on occupational risks (52.8%; *n* = 75). Regarding the employment relationship, 93% (*n* = 132) were employees of outsourced companies and 7% (*n* = 10) were government employees. According to the characteristics of the menu preparations and/or the number of meals, there was no standard dimensioning of food handlers for schools.

**Table 3:** In percentages and numbers, the sociodemographic and occupational characteristics of food handlers (Bahia/Brazil).

	**Characteristics**		**%**	***n***
Socioeconomic	Gender	Female	98.6	140
		Male	1.4	2
	Age	24–39 y/o	23.9	34
		40–49 y/o	33.1	47
		≥50 y/o	43.0	61
	Educational level	Elementary school	49.3	70
		≥High school	50.7	72
	Income—minimum wage (MW^*^)	Up to 1 MW	88	125
		More than 1 MW	12	17
Occupational	Public Servant	Yes	7	10
		No	93	132
	Time working on scholar food service	Up to 5 years	50.7	72
		More than 5 years	49.3	70
	Weekly working day	Up to 30 h	70.4	100
		40 h	29.6	42
	Participated in training on Occupational Risk	Yes	47.2	67
		No	52.8	75

* *MW, Minimum Wage in Brazil (2019)—R$ 1,100 (about U$ 275)*.

Table 4 presents the association between participation in training on Occupational Risk and the demographic and work variables of food handlers. There was no statistically significant association between participation in training on occupational risk and sociodemographic and occupational variables of food handlers.

**Table 4:** Association between participation in training on occupational risk and sociodemographic and work characteristics of food handlers.

**Variable**		**Participation in training on occupational risk**				***p*** -value^**^
		**No (%)**	**(*n =* 75)**	**Yes (%)**	**(*n =* 67)**	
Age	24–39 y/o	44.1	15	55.9	19	
	40–49 y/o	63.8	30	36.2	17	0.162
	≥50 y/o	49.2	30	50.8	31	
Educational level	Elementary school	55.7	39	44.3	31	0.495
	≥High school	50.0	36	50.0	36	
Income (mean wage—MW^*^)	Up to 1 MW	53.6	67	46.4	58	0.612
	More than 1 MW	47.1	8	52.9	9	
Public servant	Yes	50.0	5	50.0	5	0.853
	No	53.0	70	47.0	62	
Time working on scholar	Up to 5 years	54.2	39	45.8	33	0.744
food service	More than 5 years	51.4	36	48.6	34	
Intermittent Employment Contract	No	54.0	54	46.0	46	0.663
	Yes	50.0	21	50.0	21	

* *MW, Minimum Wage in Brazil (2019)—R$ 1100 (about U$ 275)*.

** *Pearson Chi-square test*.

### Anthropometric Assessment of Food Handlers and Presence of Comorbidities

The anthropometric assessment of food handlers identified mean body weight of 77.5 kg (SD = 14.7 kg), height of 159 cm (SD = 6.4 cm), WC for females of 95.56 cm (SD = 12.78 cm); for males, WC was 86.1 cm (SD = 5.72 cm) and mean BMI was 30.2 kg/m^2^ (SD = 5.38 kg/m^2^). The anthropometric evaluation of the handlers identified that 0.7% (*n* = 1) were underweight, 16.9% (*n* = 24) were normal weight, 36.6% (*n* = 52) were overweight and 45.8% (*n* = 65) were obese. Table 5 shows the association between BMI and sociodemographic, occupational characteristics, and comorbidities of food handlers. There was a statistically significant association between BMI above 29 kg/m^2^ (Overweight) and the presence of arterial hypertension and edema.

**Table 5:** Mean and standard deviation (SD) of body mass index (BMI) and association of sociodemographic and occupational characteristics and comorbidities of food handlers.

**Variables**	**Cathegories**	**Mean BMI**	***n***	**SD**	***p*** **-value**
Age	24–39 y/o	30.17	34	6.08	
	40–49 y/o	30.22	47	4.76	0.998^**^
	≥50 y/o	30.15	61	5.51	
Weekly working day	Up to 30 h	30.26	100	5.37	0.794^*^
	40 h	30.00	42	5.46	
Time working on scholar food service	Up to 5 years	30.16	72	5.88	0.966^*^
	More than 5 years	30.20	70	4.85	
Did you have health problems	No	30.20	69	5.17	0.971^*^
After joining your working role?	Yes	30.17	73	5.61	
Musculoskeletal problems	No	29.72	56	4.51	0.381^*^
	Yes	30.48	86	5.88	
Hypertension	No	29.54	93	5.44	0.049^*^
	Yes	31.40	49	5.10	
Diabetes mellitus	No	30.17	124	5.26	0.956^*^
	Yes	30.25	18	6.32	
Body edema	No	29.37	94	5.15	0.011^*^
	Yes	31.78	48	5.50	
Headaches	No	30.28	94	4.96	0.767^*^
	Yes	29.99	48	6.17	

* *Student t-test;*

** *One-way Anova*.

Food handlers answered the Perception of Exposure to Occupational Risk at SFS. The statements that obtained the lowest averages were related to the use of personal protection equipment (PPE): “I receive ear protectors to protect my ears from SFS noises” (Average = 1.1; SD = 0.67); “I get steel mesh gloves to handle meat, fish and chicken” (Mea*n* = 1.3; SD = 0.91). The highest averages were identified for the statements related to environmental comfort measures and symptoms “the noise from SFS equipment interferes with my work” (Average = 3.9; SD = 1.56); “I have headaches because of the SFS heat” (Average 3.9; SD = 1.60) and “the SFS noise gives me headaches” (Average 4.2; SD = 1.42). The final mean was 3.15 (SD = 0.69).

The percentage distribution of the classification of Perception of Exposure to Occupational Risk by handlers: 0.7% (*n* = 1) presented Very Low Perception (0 to 1.5); 20.4% (*n* = 29) had Low Perception (1.51 to 2.5); 41.5% (*n* = 59) had Fair Perception (2.51 to 3.5) and 37.3% (*n* = 53) had Good Perception (3.51 to 4.5).

Table 6 presents the association between the SFS health risk and occupational risk classification variables and the demographic, work, and comorbid characteristics with the average perception of exposure to the occupational risk of food handlers. There was a statistically significant association between handlers who presented the highest average of Perception of Exposure to Occupational Risk (2.51 to 3.5) with the identification of SFS Regular Health Risk and Regular/High Occupational Risk classification (51 to 75% adequacy; 26 to 50% adequacy, respectively). The handlers who worked in SFS classified as High Health Risk (26 to 50% of adequacy) and Very High Occupational Risk had lower Perception of Exposure to Occupational Risk scores.

**Table 6:** Health Risk and Occupational Risk classification variables of the scholar food services and the demographic characteristics, work, comorbidities with the mean of the food handlers' Perception of Occupational Risk Exposure, number of workers, standard deviation, and *p*-value.

**Variables**	**Perception of Occupational Risk Exposure**	***n***	**SD**	***p*** -value^*^
**School food service occupational risk classification**				
Very high	2.95	41	0.64	0.025
Regular/high	3.24	101	0.70	
**Weekly working day**				
Up to 30 h	3.11	100	0.71	0.265
40 h	3.26	42	0.63	
**Contract type**				
Intermittent Contract (per period)	3.05	100	0.71	0.004
Uninterrupted contract	3.40	42	0.56	
**Time working on scholar food service**				
Up to 5 years	3.28	72	0.73	0.029
More than 5 years	3.03	70	0.63	
**Educational level**				
Elementary school	3.14	70	0.68	0.736
≥High school	3.18	72	0.70	
**Presence of musculoskeletal problems**				
No	3.43	56	0.70	<0.001
Yes	2.98	86	0.63	
**Hypertension**				
No	3.16	93	0.71	0.912
Yes	3.15	49	0.67	
**Diabetes mellitus**				
No	3.18	124	0.71	0.284
Yes	2.99	18	0.52	
**Body edema**				
No	3.32	94	0.62	<0.001
Yes	2.83	48	0.72	
**Headaches**				
No	3.31	94	0.63	<0.001
Yes	2.84	48	0.70	

* *Student t-test*.

There was a statistically significant association between the score of Perceived Occupational Risk Exposure with work characteristics, type of employment contract (0.004) and time working in SFS (*p* = 0.029), and with comorbidities/symptoms, musculoskeletal problems (0.001), presence of edema (*p* < 0.001) and headaches (*p* < 0.001). There was no association between the Perception of Occupational Risk and the variables: working hours (*p* = 0.265), education (*p* = 0.736), arterial hypertension (0.912), and diabetes mellitus (0.284) (27).

## Discussion

### Characteristics of Schools' Food Services and Identification of Exposure to Occupational Risk and Measures of Environmental Comfort: Humidity, Temperature, Noise, and Luminescence

The average number of students enrolled in the municipality public schools that participated in the study was 363.1 ± 200, with a minimum number of 76 and a maximum number of 1,211. The number of meals varied in the 159 schools because some schools offered one meal and others two or three, depending on the curriculum. The number of enrolled students shows the importance of these SFS in low-income children feeding and the importance of handlers involved in these meals production.

The schools were distributed in 11 geographic areas during data collection. For each of these geographic areas, there was a nutritionist who was responsible for supervising and inspecting the activities of the SFS managed by the municipality and also supervised the activities carried out by the food concessionaires. The 11 nutritionists are responsible for supervising and inspecting SFS. The Federal Council of Nutritionists (CFN) recommends one nutritionist to supervise every 500 schoolchildren (28); therefore, to serve students enrolled in public schools who participated in the study, a minimum of 20 nutritionists would be required. This fact interferes with the monitoring of activities carried out in the SFS for food safety and, consequently, puts the health of schoolchildren at risk. To meet the objectives of the PNAE, the SFS must offer a physical structure, equipment, and utensils in good condition and compatible with the characteristics of the meals produced. An adequate number of handlers is also necessary to produce meals with nutritional, sensory, and hygienic-sanitary quality, meeting food and nutrition security principles (28). There is no standard dimensioning of food handlers for schools according to the characteristics of the menu preparations and/or the number of meals. In this study, the mean number of handlers per SFS was 2 (minimum of 1 and maximum of 5).

The WHO suggests that the handling of raw and cooked foods occur in separate areas to avoid food contamination (29). The absence of specific areas for food handling at SFS favors food production crossing of the flow, people and waste, and, consequently, the risks related to food contamination and work accidents, involving food handlers. Cardoso et al. (30) observed that 96.6% of the SFS did not have separate areas for food preparation, and 50.2% of the schools did not have a linear and unidirectional flow due to inadequate sizing areas. This favors food contamination and puts the worker's health at risk.

Food handlers who work at SFS are responsible for all stages of meal production, from receiving foodstuffs to waste management. This excess of activities, associated with precarious physical structure, insufficient number of equipment and utensils (in inadequate conservation), favors the exposure to occupational risk. In addition to these aspects, it is essential to recognize external factors to the work environment, such as individual characteristics: age, nutritional status, and presence of comorbidities.

Souza et al. (31) carried out a literature review that included 18 studies in 391 public schools in all geographic regions of Brazil about evaluating good hygiene practices in public schools. The authors found that 54.12% of exposure to health risks resulted from SFS physical conditions (regular risk) (19). The studies included in the review reinforce the exposure of workers and their low perception of occupational risks. Therefore, it is essential that workers participate in continuing education programs to adopt practices related to food hygiene and the perception of exposure to occupational risk and have access to control measures to minimize and/or eliminate risks (32).

### Characteristics of SFS Food Handlers

As the SFS activities are associated with domestic services, the predominant presence of females is perceived (33), as occurred in this study. Cardillo, Gema, and Fuentes-Rojas (34) identified that 100% of handlers were female (seven cis women and one trans woman) when analyzing the activities of food handlers in three state schools in the city of Campinas, São Paulo/Brazil. Martins, Hogg, and Otero (35) identified that 96% of the handlers were female in a study carried out in Portugal in a company that distributes meals to schools and long-stay institutions for the elderly (ILPI).

Pagotto et al. (36) also identified that 84% of handlers were female when assessing the level of knowledge, attitudes, and practices in food handlers in 27 commercial restaurants located in the city of Vitória, Espírito Santo/Brazil. However, Araújo et al. (37) identified 62.9% of male handlers in 37 community restaurants in Braz. Moghnia et al. (38) reported 84.7% of male handlers in community restaurants and health centers in six Kuwait provinces.

The presence of most female handlers in food services is related to maternal care associated with women and the concentration of these more precarious and undervalued sectors of work (housework, cleaning, caregiver). Men are assigned the positions of command and power with better payment (39). Demographic Census (40) showed that the average monthly income of men with a formal employment relationship was R$1,392.00 (>1 minimum wage), and for females, it was R$983.00 (<1 minimum wage). This trend reinforces the devaluation of female labor in the labor market in Brazil, although women are practically half of the workforce (40).

The minimum age among the handlers in this study was 24 and the maximum was 72 years, with the age group with the highest number of professionals between 40 and 59 years old. These data are different from the study by Moghnia et al. (38), who found the largest number of handlers (77.3%) aged between 24 and 39 years, but this is similar to data from the study by Martins et al. (35), as 67% of handlers were between 36 and 55 years. In this study, these data are possibly associated with the working time of food handlers in schools.

The level of education can contribute to the formation of critical thinking and is one of the strategies for social ascension (41). However, although most handlers had completed high school in this study, most did not perceive the risk they were exposed to. This data is different from the study by Barros et al. (42), who identified a more homogeneous distribution of schooling with the presence of 15% of handlers with incomplete elementary education, 25% with complete elementary school, 17% with incomplete high school, 40% with complete high school and 3% incomplete graduation. Araújo et al. (43) and Pagotto et al. (36) also identified that 52% of handlers had completed high school. However, Moghnia et al. (38) identified complete high school and higher education levels for 43.2% of handlers. In general, because they need knowledge about cooking, food services workers are not among the youngest workers who have not yet completed high school, which can be seen in these workplaces' working time.

Regarding the time working in SFS, most worked as food handlers for up to 5 years. These data are different from another study (35) in which 64.3% had more than 5 years working as food handlers. Most of our sample did not participate in training on Occupational Risk (occupational hygiene). There was no statistically significant association between participation in training and the other sociodemographic and work variables. The training of food handlers on occupational risks is not mandatory by the Regulatory Norms and the sanitary legislation in Brazil (18).

In general, mandatory topics in handler training refer to food hygiene to implementing good manufacturing practices to produce safe food. However, training in occupational risk would allow handlers to perceive it and adopt control measures such as the use of PPE and seek better working conditions—workers' sizing. These routines do not favor work overload and activity rotation.

According to Pagotto et al. (36) only 26.7% reported not participating in occupational risks training. A similar result was seen in Cunha et al. (44), where 31.7% of handlers did not receive any training. It is important to highlight that the food handlers' training is a previously planned learning strategy to improve knowledge about the activities performed and consolidate permanent changes in practices and attitudes, including risks. Although Brazilian labor legislation does not recommend training on occupational hazards, it includes mandatory use of PPE and CPE (18).

Food handlers' nutritional status and chronic non-communicable diseases (NCDs) identification allow food services to adopt preventive measures, avoid absenteeism, and promote health. NCDs are a serious global public health problem (45). In 2012, 68% of worldwide deaths were related to NCDs (45). In Brazil, this group of diseases corresponds to ~75% of the causes of death in people aged 30 to 69 years (work productive years) (46, 47).

In this sense, the adoption of measures reducing the impact of NCDs on population disability, morbidity, and mortality has been the concern of several countries due to the expenses of such diseases. NCDs compromise the population's quality of life and represent an expected expenditure in 15 years (2011 to 2025) of around seven trillion dollars in low and middle-income societies, such as Brazil (46, 48).

In this study, the handlers' average BMI was classified as obese. The WC for females is above the World Health Organization recommendation to prevent cardiovascular diseases (48, 49). Similar data were identified by Fideles et al. (50), where 59.9% of food handlers working in 36 Popular Restaurants in Brazil were overweight/obese. A study by Simon et al. (51) with food handlers of the Clinic Hospital of Porto Alegre/Brazil observed that 60.8% of the employees were overweight (35.1%) and obese (25.7%), and 77.3% had abdominal circumference above the values considered normal.

This study showed an association between BMI above 29 kg/m^2^ (excess weight) and hypertension and body edema. Similar data were found in the studies by Fidelis et al. (50) who identified hypertension as the most prevalent NCD diagnosed in 45.8% of food handlers with NCDs, and Simon et al. (51), who showed a significant association of excess weight with systolic blood pressure in 80.76% of public hospital employees in Porto Alegre/Brazil. Consuming carbohydrate-rich foods, time in the service, absence of breaks during the workday, and a sedentary lifestyle are among the factors associated with overweight in food handlers (50, 51).

The critical issue in understanding the economic impacts of excess weight is that mortality is not the only important outcome. Decreased productivity, increasing disability, rising health care costs, early retirement, and reduced healthy living throughout life affect human capital in countries. According to Shekar and Popkin (52), the estimated economic costs of obesity vary considerably. For example, US estimates range from $89 billion to $212 billion in total costs; China estimates between 5.8 and 8.7% of the gross national product (GNP) in 2020 to 2025, respectively; and Brazil projects obesity-related healthcare costs doubling from US$5.8 billion in 2010 to US$10.1 billion in 2050.

### Perception of Exposure to Occupational Risk

The perception of exposure to occupational risks showed that the handlers do not give importance to using PPE as a practice to minimize exposure to occupational risk. Those with the highest scores were related to measures of environmental comfort: temperature and noise, associated with headaches and interference in carrying out activities. However, all measures were under the Brazilian legislation (13, 18, 21–24, 27, 53).

The study identified that handlers who worked in SFS with very high occupational risk and high health risk had the lowest average perception of exposure to occupational risk. This is possibly due to the inexistence of risk perception or the incapacity to perceive the risk, claim improvements, or leave the activity. The country's unemployment situation and the precarious employment from the labor reform adopted in Brazil in 2017 (54) may relate to this inexistence of risk perception. The Brazilian labor reform jeopardized the access to social security benefits and funding sources, given the growing disruption of the labor market and the introduction of more unstable and precarious hiring forms, such as self-employment, outsourcing, and intermittent contracts (55). In addition, females also face significant barriers concerning employment opportunities, even with higher education. They receive job offers with lower wages, which shows that unemployment is also associated with issues of gender discrimination or jobs related to domestic care or with low pay. The uncertainties generated by the new processes and precarious work links and the fear of unemployment itself result in experiences of insecurity, vulnerability, and social suffering. Regarding the hygiene and health of handlers, Lemos et al. (56) found that in 60% of the SFS, there was no health control program for handlers, and Ferro et al. (57) found that 55.9% of handlers from 35 public schools in the state of Tocantins/Brazil did not perform regular health exams. It is a sanitary requirement to prevent the spread of foodborne diseases during food handling that food handlers have their regular exams (18). The WHO recommends adopting sanitary practices by food handlers to protect consumers. Among these are the performance of regular examinations: physical, medical histories, oropharyngeal culture, blood count, x-rays, skin and stool cultures for the detection of symptomatic or asymptomatic carriers (58).

### Study Limitation

The study was limited to SFS in Bahia/Brazil, and it does not represent the entire country. The cross-section nature of this study is a potential limitation since correlational results do not reveal causal relationships between the variables. Also, most participants were female, but the nature of services in SFS can explain it.

## Conclusion

Food handlers from SFS in Bahia/Brazil are primarily female with <5 years of working in the schools and no training on occupational risks. This lack of training is not associated with demographic or other occupational variables. The majority of the food handlers present a fair or good perception of exposure to occupational risk. The lowest average related to the perception of exposure to occupational risk was PPE use, and the highest was related to environmental comfort measures. These food handlers are also mostly overweight, and higher BMI was associated with hypertension and edema. The workers of public schools in Bahia/Brazil are, like other workers linked to the PNAE, exposed to health risks and occupational risks related to health risks. If these workers do not perceive themselves exposed to such risks, there is no way to protect themselves. Therefore, the exposure conditions must be reduced by complying with the sanitary legislation that contemplates the ideal working conditions in these SFS. In addition, workers must be trained and informed about the ideal working conditions. However, it is difficult to receive proper training and information with the low number of nutritionists that support the studied schools. The precariousness of the work of food handlers also involves the reduction of unemployment in Brazil and gender policies that seek equal pay and working conditions for women.

## Data Availability Statement

The original contributions presented in the study are included in the article/supplementary material, further inquiries can be directed to the corresponding author/s.

## Ethics Statement

This study is part of a broader research project entitled Occupational Risks in School Food Services, approved by the Research Ethics Committee of the School of Nutrition of the Federal University of Bahia (protocol n° 2,121,882). The patients/participants provided their written informed consent to participate in this study.

## Author Contributions

JF, MA, and RA: conceptualization, methodology, formal analysis, and investigation. JF, MA, RA, and EN: validation. RZ: resources. JF: data curation. JF, RA, RZ, and RB: writing—original draft preparation. JF, RA, RZ, AR, and RB: writing—review and editing. JF, MA, RA, RZ, AR, and RB: visualization. RA, MM, and AA-M: supervision. JF, AR, and HH: project administration. All authors have read and agreed to the published version of the manuscript.

## Conflict of Interest

The authors declare that the research was conducted in the absence of any commercial or financial relationships that could be construed as a potential conflict of interest.

## Publisher's Note

All claims expressed in this article are solely those of the authors and do not necessarily represent those of their affiliated organizations, or those of the publisher, the editors and the reviewers. Any product that may be evaluated in this article, or claim that may be made by its manufacturer, is not guaranteed or endorsed by the publisher.
